# Hierarchical Oil–Water–Oil Pickering Double Emulsions Stabilized by Tubular Nanoparticles

**DOI:** 10.1021/jacs.6c01861

**Published:** 2026-04-06

**Authors:** Ludovico Guercio, Chiara Ferlito, Giulia D’Agostino, Lorenzo Lisuzzo, Giuseppe Cavallaro, Giuseppe Lazzara, Konstantin Dolgan, Yuri M. Lvov

**Affiliations:** † Department of Physics and Chemistry − E. Segrè, Università degli Studi di Palermo, Viale delle Scienze, pad. 17, 90128, Palermo, Italy; ‡ 5160 Louisiana Tech University, Institute for Micromanufacturing, Ruston, Louisiana 71272, United States

## Abstract

Pickering emulsions stabilized by a small fraction of submicrometer-size clay tubes provide stable and tunable soft-matter systems with relevance across multiple fields. We here introduce a novel oil-in-water-in-oil Pickering double emulsion system stabilized by selectively functionalized halloysite nanotubes. The stability of the double emulsions was achieved through the combined use of pristine and surface-modified halloysite nanotubes, which selectively stabilized the oil-in-water and water-in-oil interfaces in the two separately prepared colloids. After these two samples were mixed and stirred, a hierarchical structure of oil microdroplets inside water droplets dispersed in bulk oil was obtained. Fluorescent microscopy and particle-tracking analyses confirmed that oil droplets confined within the double emulsion exhibit Brownian motion with decreased diffusion coefficients. The resulting dual-compartment structure enables the simultaneous encapsulation of hydrophilic and hydrophobic species, promising applications in controlled delivery and multifunctional formulations.

## Graphical abstract

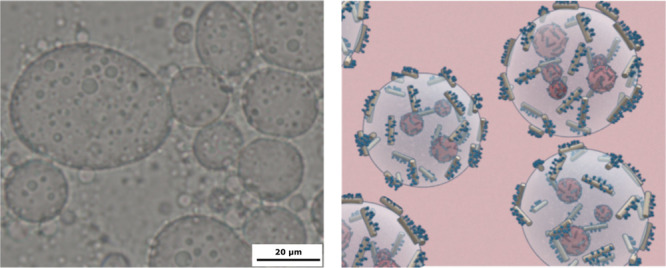

A Pickering emulsion is interfacially stabilized by a small amount of solid micro- or nanoparticles rather than conventional surfactants.^1,2^ A simple preparation, high emulsion stability, narrow droplet size distribution, and good biocompatibility make them attractive for both fundamental research and practical applications.^3–5^ Due to the new challenges in various technologies, the interest in this type of systems has increased.^6,7^ Pickering emulsions can be tailored to desired applications by tuning the physicochemical properties of the stabilizing particles, allowing precise control over the emulsion type (oil-in-water or water-in-oil), droplet morphology, and rheological behavior.^8,9^ Their versatility can be further enhanced by introducing an additional phase, enabling the formation of more complex architectures such as hierarchical multiple emulsions by development of double Pickering systems, displaying droplet-in-droplet architectures with internal and external interfaces stabilized by solid particles.^10,11^ Designing such structures requires a precise selection of emulsifiers, whose distinct wettability should allow for the stabilization of either the oil-in-water or the water-in-oil phases.^12,13^ The dual-compartmented architecture makes these promising for stimuli-responsive delivery systems, microreactors in catalysis and the design of advanced functional materials where storage, protection and sequential release of active species is required.^14–16^ The Pickering double emulsion systems are gaining increased attention in environmental remediation, due to their interfacial stability and tunable surface chemistry, which enable efficient adsorption, capture, and controlled removal of pollutants and hydrophobic contaminants.^17^

In this work, we describe a new class of Pickering double emulsions prepared with natural clay nanotubes whose wettability was tuned by selective functionalization in order to stabilize either the oil-in-water (o/w) or water-in-oil (w/o) emulsions. Halloysite oil-in-water and water-in-oil Pickering emulsions were prepared separately and then mixed forming a stable three-level oil–water–oil system, as shown in Scheme 1.

**Scheme 1:**
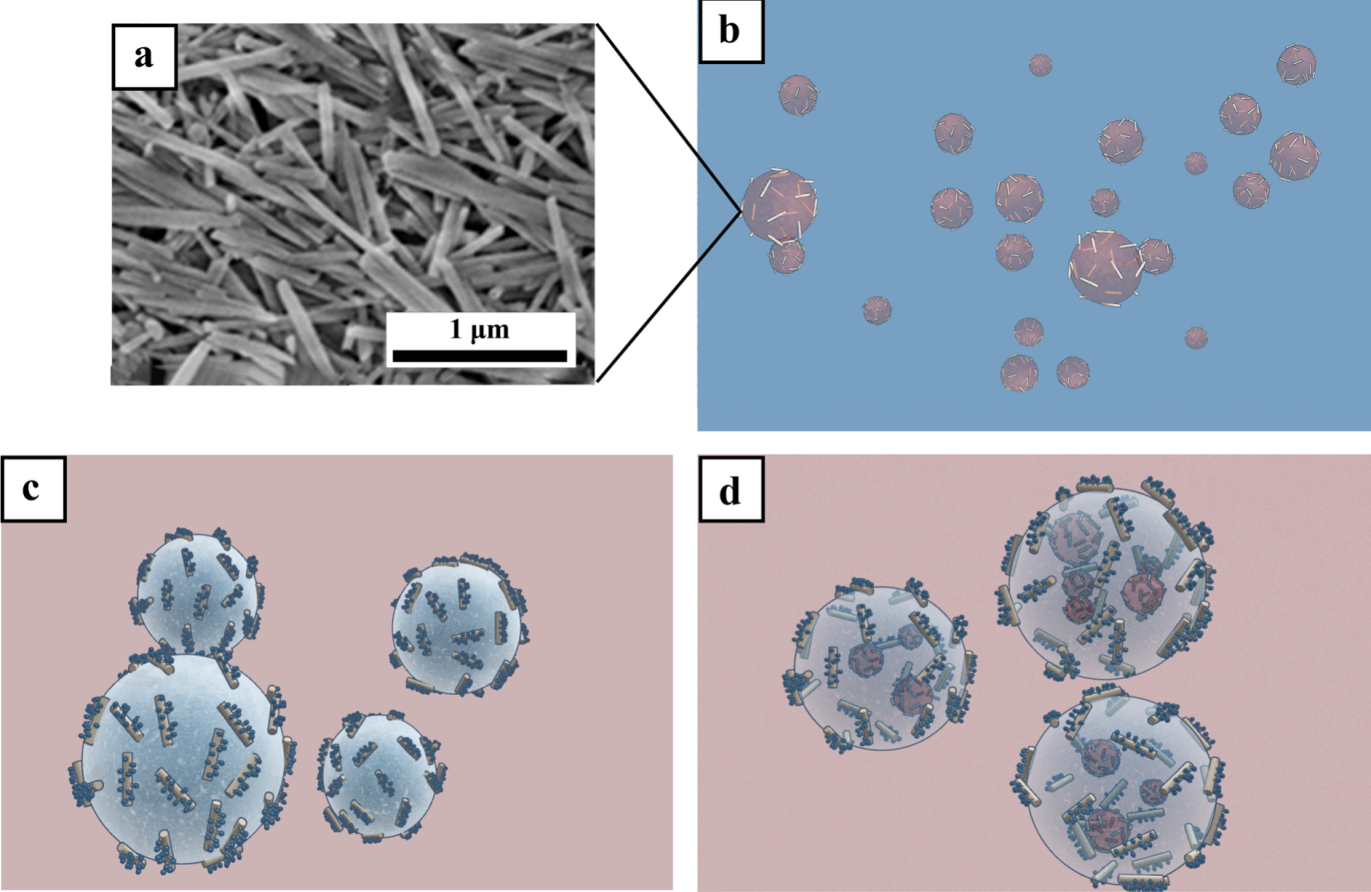
(a) SEM Image of Pristine Clay Nanotubes; Schematic Representation of (b) o/w Pickering Emulsions Stabilized by Pristine HNTs, (c) w/o Pickering Emulsions Stabilized by Functionalized HNTs, and (d) o/w/o Pickering Double Emulsions System (Oil Droplets Inside Water Droplets Inside Bulk Oil)

Halloysite nanotubes (HNTs) are natural clay minerals which possess a hollow tubular morphology composed of aluminosilicate layers rolled into a spiral-like structure.^18–20^ Their peculiar chemical properties arise from the different chemical compositions of the inner and outer surfaces.^21,22^ Indeed, the external surface carries a negative charge, originating from tetrahedral SiO_4_ sheets, while the inner lumen, formed by octahedral AlO_3_(OH)_3_ layers is positively charged.^23,24^ The charge separation, combined with the tubular architecture, allows for controlled and targeted surface modifications, making halloysite a good candidate for the design Pickering emulsions.^25–27^ Due to the dynamic nature of the proposed multiinterfacial architecture, achieving a stable double emulsion system requires precise control over the wettability of the nanotubes.

Therefore, to encapsulate the water-in-oil phase, pristine p-HNTs were partially coated with cationic alkyltrimethylammonium bromide on the outer negative surface, leading to the formation of functionalized more hydrophobic f-HNTs.^28^ The optimized functionalization of the nanotubes was confirmed using the following characterization techniques: dynamic light scattering and zeta-potential measurements revealed an increase in both particle size and surface charge, indicating the effective modification of the nanotube surface but preventing their fast precipitation in water (). This was further confirmed by contact angle measurements, which showed a change in wettability before and after the treatment (the details of the procedures are provided in the Supporting Information (SI)).

The core of this work is the functionalization of solid tubule nanoparticles to tailor surface chemistry and wettability, enabling the selective stabilization of oil–water interfaces. Beyond halloysite nanotubes, particles with different compositions and morphologies can be explored within the same framework. For example, graphene oxides can act as Pickering emulsifiers due to their tunable surface functionalities, and carbon nanotubes stabilize either o/w or w/o emulsions with their elongated morphology reducing droplet coalescence.^29^ More general, other inorganic or hybrid nanoparticles with controllable surface functionalization may extend this strategy, broadening the range of stabilizers and enhancing the versatility of complex emulsion architectures.

The protocol for the design of the Pickering double emulsions involves the separate preparation of oil-in-water (o/w) and water-in-oil (w/o) emulsions, which are stabilized by p-HNTs and f-HNTs, respectively. In both cases, decane was used as the oil phase. The preparation of both o/w and w/o Pickering emulsions was investigated by systematically varying the composition range prior to formulating the double o/w/o system (see Materials and Methods in SI). The concentrations selected for this study were those identified as optimal, as they ensured a high yield of well-formed Pickering emulsions suitable for further investigation while preventing the formation of dense clay aggregates that would hinder processing and analysis.

The water/decane volume ratios for the o/w and the w/o emulsions were 60:40 and 40:60 respectively. After sonication, the aqueous portion of the first sample containing the o/w emulsions was transferred to the equal volume of the oil phase of the second w/o emulsion. The resulting mixture was stirred for 5 min, resulting in the Pickering halloysite based double emulsions: a hierarchical system consisting of bulk oil containing 40–80 μm water droplets that encapsulate smaller 2–4 μm oil droplets.

Figure 1 displays microscopy images of the systems: (a,b) o/w emulsion and (c,d) w/o emulsion. Panels (e,f) and, at higher magnification, panels (g,h) show the o/w/o double emulsion obtained after mixing the two initial samples (macroscopic photos are shown in Figure S2). The use of hydrophilic p-HNTs, with a contact angle of 30°, leads to the formation of stabilized oil microdroplets, as confirmed by optical and fluorescence microscopy using the lipophilic fluorescent dye Nile Red (excitation range 460–500 nm), Figure 1b. The oil forms the core of the p-HNT stabilized droplets. These results confirm formation of HNTs stabilized oil-in-water emulsions, as also was demonstrated for petroleum spill emulsification in seawater.^30^

**Figure 1:**
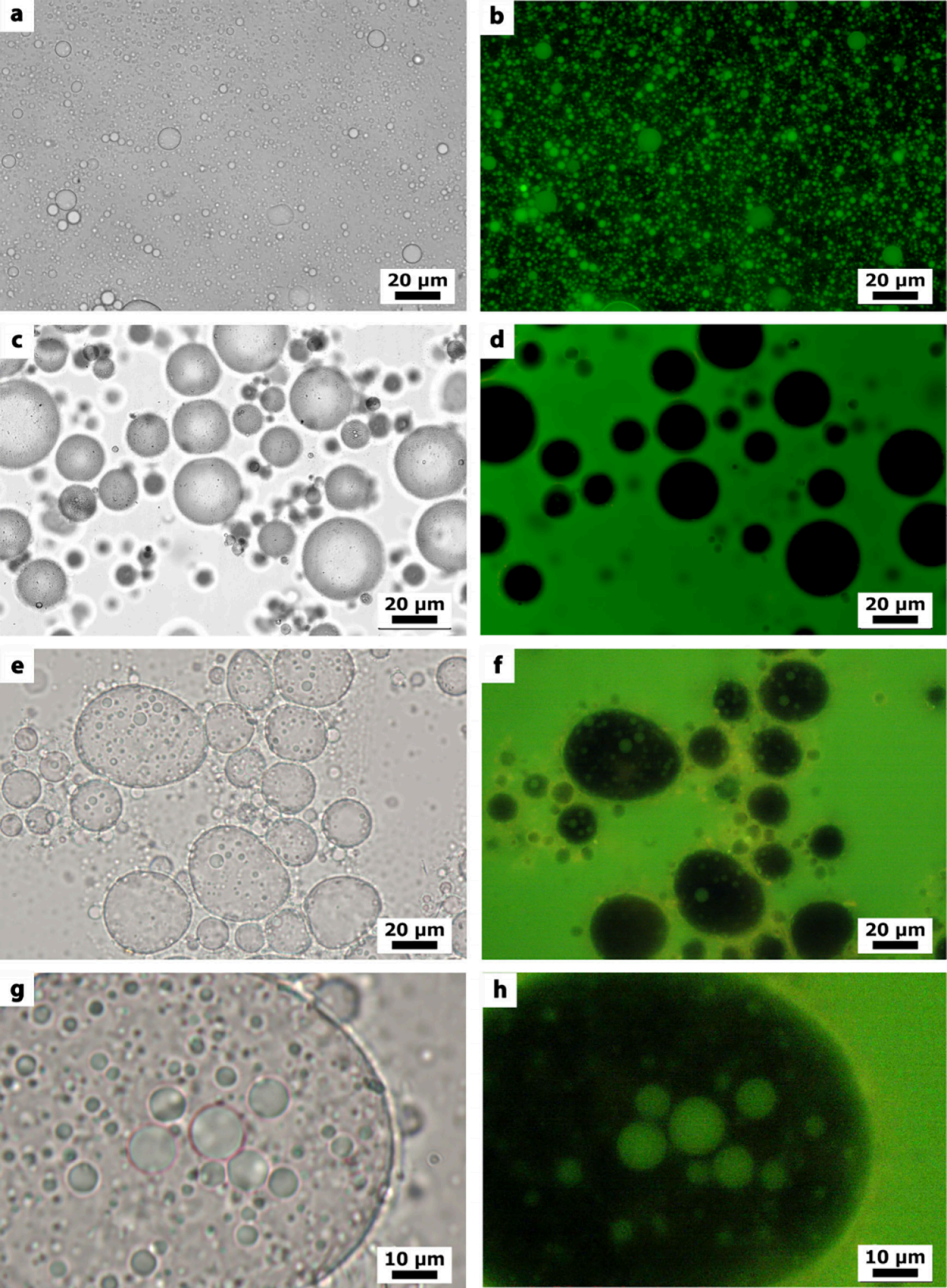
Optical microscopy images of (a) o/w Pickering emulsions stabilized by p-HNTs; (c) w/o Pickering emulsions stabilized by f-HNTs; (e) o/w/o Pickering double emulsions at 40× and (g) 60× magnifications. (b–h) Corresponding fluorescence images obtained using Nile Red as a probe; green is oil, and black is water.

Similarly, the w/o droplets were observed when hydrophobized f-HNTs with a contact angle of 101° were used as emulsifiers (Figure 1c,d). In this case, fluorescence microscopy proves the water-in-oil emulsion, as no Nile Red interacts with its core but shows green in the continuous oil phase.

The stabilized oil or water droplet dimensions are affected by the process: o/w emulsions stabilized by p-HNTs are much smaller in diameter than the w/o emulsions stabilized by f-HNTs. Finally, the two systems were mixed, and the Pickering double emulsion was formed (Figure 1e–h). Fluorescence microscopy allows visualization of their hierarchical structure: smaller o/w droplets are confined within the cores of larger w/o Pickering emulsions, demonstrating that an oil-in-water-in-oil (o/w/o) Pickering double emulsion was successfully prepared by using clay nanotubes (Movie S1, Supporting Information). The thermal and the time-dependent stability of the Pickering double emulsions were investigated. In particular, the emulsions remained stable up to 50 °C. At this temperature, droplet coalescence began to occur, while complete phase separation was observed at 60 °C (Figure S3). Regarding time stability, complete phase separation was detected after 24 h (Figures S4–5). Most importantly, in both cases, the system could be restored. After cooling the heated sample to room temperature and upon rapid remixing, even after time-induced separation, the o/w/o Pickering double emulsions reformed and became clearly visible again. Therefore, both the thermally induced and the time-dependent destabilization processes are reversible (the details are reported in the SI).

The analysis of the droplet motion and their diffusion coefficients is illustrated in Figure 2. Any differences arising in the movement of o/w droplets were studied under two conditions: (i) Single o/w Pickering emulsions when the oil emulsions are dispersed in the continuous water phase, and (ii) o/w emulsions when they are confined within the double o/w/o Pickering system. For both systems, diffusion coefficients were determined through particle-tracking analysis. Video frames were processed to enhance the visibility of the selected particles, which were approximated as spherical for the trajectory extraction. The diffusion behavior of a spherical particle can be described by the translational diffusion coefficients along two dimensions (D_*x*_, D_*y*_). The experimental diffusion coefficients were calculated by the slopes of the mean squared displacements of the particle as a function of the lag time.^31,32^

$$MSD_{x}+MSD_{y}=\left\langle {\Delta x}^{2}+{\Delta y}^{2}\right\rangle =2\left(D_{x}+D_{y}\right)\Delta t$$

Displacements were measured in the lab frame (*x*, *y*); Δ*t* is the lag time.

**Figure 2:**
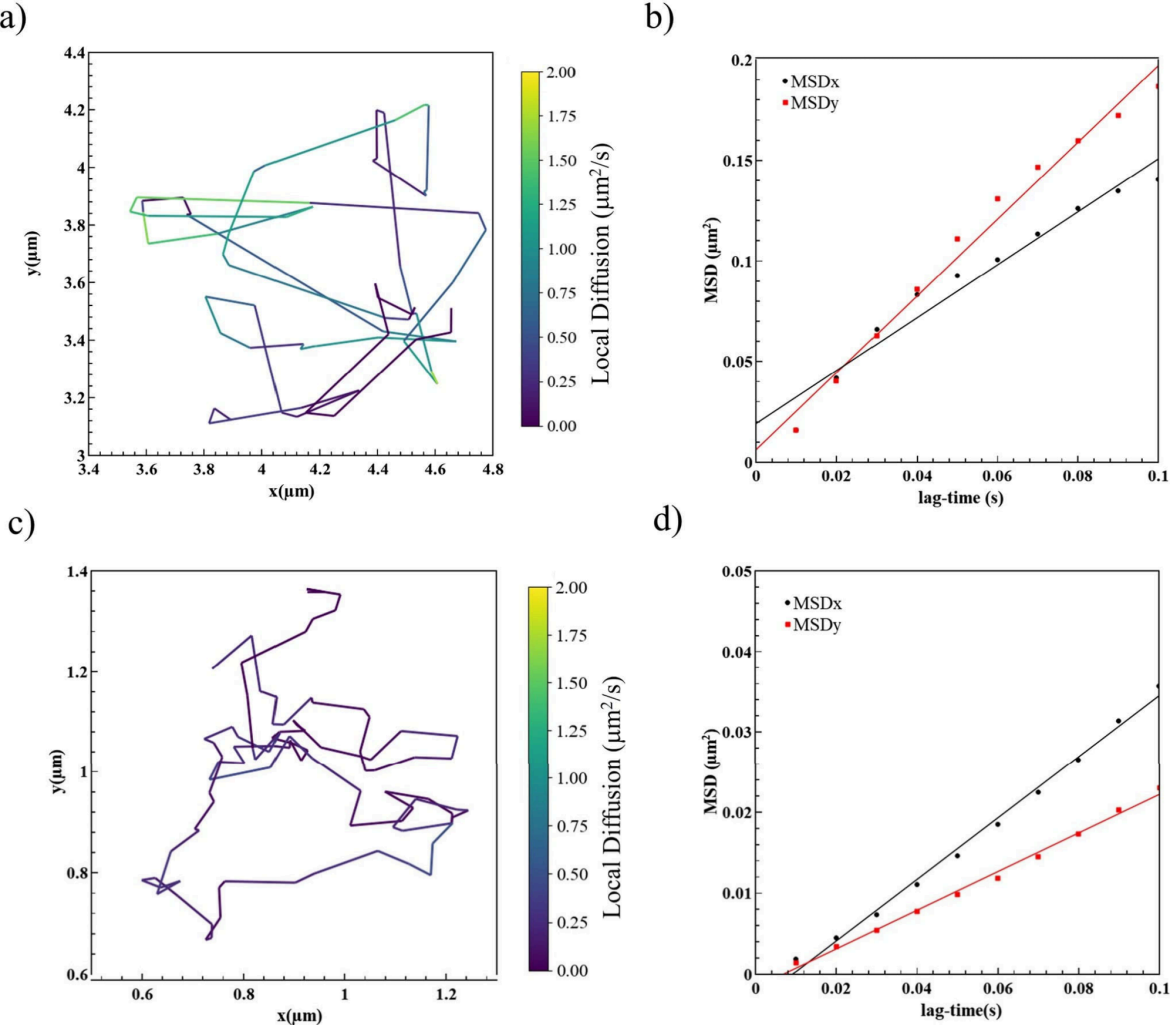
(a,b) Tracking of oil microdroplets and their mean squared displacements (MSD) along the *x* and *y* axes in the bulk o/w systems and (c,d) in confined o/w/o systems (double Pickering emulsion). The color bars in (a,c) indicate their local diffusion rate.

The resulting trajectories of the particles (Figure 2a) suggest that the free o/w Pickering emulsions exhibit an overall Brownian motion. Specifically, their diffusion along the axes is *D*_*x*_ = 0.95 μm^2^ s^–1^ and *D*_*y*_ = 0.6 μm^2^ s^–1^ (Figure 2b). In the case of the tiny inner oil droplets double confined within the aqueous phase of o/w/o emulsions, they also display Brownian motion in the surrounding water space but with a lower average velocity (Figure 2c). The measured diffusion coefficients are *D*_*x*_ = 0.16 μm^2^ s^–1^ and *D*_*y*_ = 0.12 μm^2^ s^–1^ which are much less than in the first o/w case. The droplets within the o/w/o double emulsion diffuse five times more slowly than the simpler o/w system, indicating that their motion is more restricted. In this double emulsion, tiny oil droplets reside inside the inner part of the aqueous droplets and are themselves suspended in the bulk oil phase. As a result, the maximum local diffusion rates are 1.8 and 0.5 μm^2^ s^–1^ in the two cases, the lower speed being due to the reduced available volume for particles movement and to more frequent collisions with droplet boundaries.

In conclusion, a new type of Pickering double emulsion with an oil/water/oil hierarchy based on natural clay nanotubes was developed and characterized. Due to its dynamic nature and structural versatility, this hierarchical system serves as a model for heterogeneous materials. Its compartmentalized architecture, which was preliminarily demonstrated by the controlled release of a hydrophobic dye from the Pickering double emulsions (see SI), enables spatial separation and coexistence of different chemical environments, making it particularly attractive for catalysis, where incompatible catalysts or sequential reactions can be integrated within a single platform. The simultaneous encapsulation of hydrophobic and hydrophilic species provides opportunities for wastewater treatment and drug delivery, allowing for the efficient capture, protection, and controlled release of active compounds. Further potential exists in cultural heritage conservation, where cleaning agents can be loaded into separate oil phases to achieve controlled, broad-spectrum cleaning with minimal impact on delicate surfaces. It is also possible to design double emulsions with a plethora of other emulsifiers possessing different morphology. The suggested approach can be extended for architectures with an even higher level of compartmentalization, such as the preparation of Pickering triple emulsions.

## Supplementary Material





## Notes

Movie S1. Optical microscopy of Pickering double emulsions (MP4)
Detailed experimental procedures including halloysite nanotubes functionalization, Pickering emulsions preparation, characterization methods; additional experiments and discussions (PDF)
