# Supplementary material for: Hierarchical Oil–Water–Oil Pickering Double Emulsions Stabilized by Tubular Nanoparticles
Source: J Am Chem Soc. 2026 Apr 6;148(14):14726–31. doi: 10.1021/jacs.6c01861 (PMC13088240; doi:10.1021/jacs.6c01861)
Supplement: Supplementary file 2 [file ja6c01861_si_002.pdf]

Supporting Information

Hierarchical Oil-Water-Oil Pickering Double Emulsions Stabilized by Tubular Nanoparticles

Ludovico Guercio [a], Chiara Ferlito [a], Giulia D'Agostino [a], Lorenzo Lisuzzo [a,] Giuseppe Cavallaro [a], Giuseppe Lazzara [a], Konstantin Dolgan [b], Yuri M. Lvov [b,*]*

[a] Department of Physics and Chemistry – E. Segrè, Università degli Studi di Palermo, Viale delle Scienze, pad. 17, 90128 Palermo, Italy. *lorenzo.lisuzzo@unipa.it
[b] Louisiana Tech University, Institute for Micromanufacturing, Ruston, Louisiana 71272, United States. *ylvov@latech.edu

This PDF file includes:

-Materials and Methods

Materials

Surface modification of Halloysite Nanotubes

Preparation of o/w Pickering emulsions

Preparation of w/o Pickering emulsions

Preparation of Pickering double emulsions

Release of Nile red from Pickering double emulsions

Methods

- Characterization and Experimental Details

DLS, ζ -potential, wettability of pristine and functionalized halloysite nanotubes

Macroscopic and fluorescence microscopic images Pickering emulsions

Effect of the temperature on the o/w/o Pickering double emulsions

Stability of the o/w/o Pickering double emulsions as a function of time

Release of Nile Red from o/w/o Pickering double emulsions

-Supporting References

MATERIALS AND METHODS

Materials. Halloysite nanotubes (HNTs), hexadecyltrimethylammonium bromide (C16Br), decane and Nile red ($C_{20}H_{18}N_2O_2$ – MW: 318.37 $gmol^{-1}$) were purchased from Sigma-Aldrich and used as received, without further purification.

Surface modification of Halloysite Nanotubes. The hydrophobization of halloysite nanotubes was carried out as reported in literature.[1,2] Firstly, an aqueous solution of hexadecyltrimethylammonium bromide (C16Br) was prepared by dissolving 2 g of the surfactant in 250 g of deionized water under stirring until a clear solution was obtained. Then, 4 g of HNTs were added and the dispersion was stirred continuously for 48 h to allow adsorption of the surfactant onto the clay surface. The resulting system was centrifuged at 7000 rpm to recover the functionalized halloysite. The collected solid was washed repeatedly with deionized water until surface tension measurements of the supernatant indicated the complete removal of unbound species. Finally, the powder was dried at 80 °C and stored in a desiccator at 25 °C until further use.

Preparation of o/w Pickering emulsions. Oil-in-water Pickering emulsions were prepared by introducing 1.5 mL of water and 1.0 mL of decane into a glass vial. Subsequently, pristine halloysite nanotubes were added at a concentration of 0.5 wt%. Below this value, very low amounts of Pickering emulsions were obtained whereas, at higher concentrations, the emulsions could still be prepared, but the high excess of clay led to slurry samples, with major issues on further studies.

After the addition of the clay, the system was stirred at 600 rpm for 5 minutes and then sonicated for additional 5 minutes. The emulsion was sampled from the aqueous bulk phase for further characterization.

Preparation of w/o Pickering emulsions. Water-in-oil Pickering emulsions were prepared by adding 1.5 mL of decane and 1.0 mL of water into a glass vial, followed by the addition of 0.7 wt% functionalized halloysite nanotubes. Also in this case, very low amounts of Pickering emulsions were obtained with smaller concentration of f-HNTs whereas, for higher amounts, the emulsions could still be prepared, but the high excess of clay led to slurry samples, with major issues on further studies. Then, the mixture was stirred at 800 rpm for 5 minutes, after which the emulsion was directly analyzed by optical microscopy.

Preparation of Pickering double emulsions. Finally, for the oil-in-water-in-oil (o/w/o) double emulsions, the aqueous bulk phase from the o/w emulsion and the oil bulk phase from the w/o emulsion were combined in a new glass vial. The resulting mixture was stirred at 400 rpm for 5 minutes to form the double emulsions system. The system was subsequently characterized after collecting the samples from the oil phase for further analysis.

Release of Nile red from Pickering double emulsions. The study of the Nile red release kinetics was carried out via UV–VIS spectrophotometry. Ethanol was used as releasing medium. Specifically, 2 mL of ethanol were added to a quartz cuvette together with 0.5 mL of the o/w/o Pickering double emulsions containing Nile Red at a concentration of 4μg mL^{-1}. The absorbance values at 550 nm were considered and spectra were recorded as a function of time while the sample was left under gentle stirring. The same experiment was conducted by adding 0.5 mL of dye solution in decane to 2 mL of ethanol for comparison.

Methods. ζ-potential and Dynamic Light Scattering (DLS) measurements were performed by using a Zetasizer Nano-ZS (Malvern Instruments) under isothermal conditions (T = 25 °C ± 0.1) to

investigate both HNTs and f-HNTs samples. Aqueous dispersions were prepared at 10^{-3} wt%. DLS measurements were performed at a scattering angle of 173° and a wavelength of 632.8 nm. The field-time autocorrelation functions were analyzed by ILT to obtain the distribution of the apparent hydrodynamic radii. Water contact angle analysis was carried out by using an optical device (OCA 20, Data Physics Instruments), equipped with a high-resolution CCD camera and a high-performance digitizing adapter. Data were acquired using the SCA 20 software (Data Physics Instruments). The sessile drop method was used by the deposition of a droplet (12.0 ± 0.5 µL) on the samples surface. The measurements were conducted at 25.0 ± 0.1 °C. Field Emission Scanning Electron Microscopy images were taken using a Hitachi S-4800 (Japan) instrument. Before imaging, samples were decorated with 3 nm gold. Optical microscopy images were acquired using a NeXcopeNE710 microscope (TiEsseLab) equipped with a digital camera (TiEsseLab MIchrome 20) and implemented with a multi wavelength LED source (NeXcope FL 900C LED) to perform fluorescence microscopy. For this purpose, 50 µL of Nile red dye were added to 0.5 mL of the emulsion samples and gently mixed before observation. The excitation and emission wavelengths for Nile red are 460-500 nm and 539 nm, respectively. A statistical analysis on the dimensions of the prepared samples as a function of time was conducted using ImageJ as software.[3]

For particles motion analysis, video acquisition was performed at a rate of 100 fps. The particle centre of mass (*x, y*) was tracked using the open-source software ImageJ 1.54p. Images were first processed by adjusting contrast and brightness to clearly highlight the regions of the Pickering emulsion without optical interference.[4] The release profiles of Nile red were studied by UV–Vis spectrophotometer (Specord S600 Analytik Jena).

Characterization of pristine and functionalized HNTs

After the functionalization protocol of halloysite nanotubes was carried out, DLS measurements were conducted to study any change in the size of the sample. **Figure S1** reports that the f-HNTs have larger diameters than the corresponding p-HNTs, due to the attachment of the surfactants on the outer surface. Also, it is worth to note that the zeta potential is heavily affected by the modification of the surface (Figure S1b). Indeed, the values increased from −26.5 to +42.4 mV after the grafting step was carried out. These results can be explained by taking into account the partial screening of halloysite negative charges on the outer Si based surfaces due to the interactions with the hexadecyltrimethylammonium bromide. Hence, aimed at assessing the wettability of the samples, contact angles were considered. As it is reported in Figure S1c, p-HNTs are very hydrophilic and their contact angle is 30°. Conversely, f-HNTs become highly hydrophobic nanoparticles, with a contact angle of 101°. In light of this, it is possible to state that the modification of HNTs outer surface with C16Br was successfully conducted.

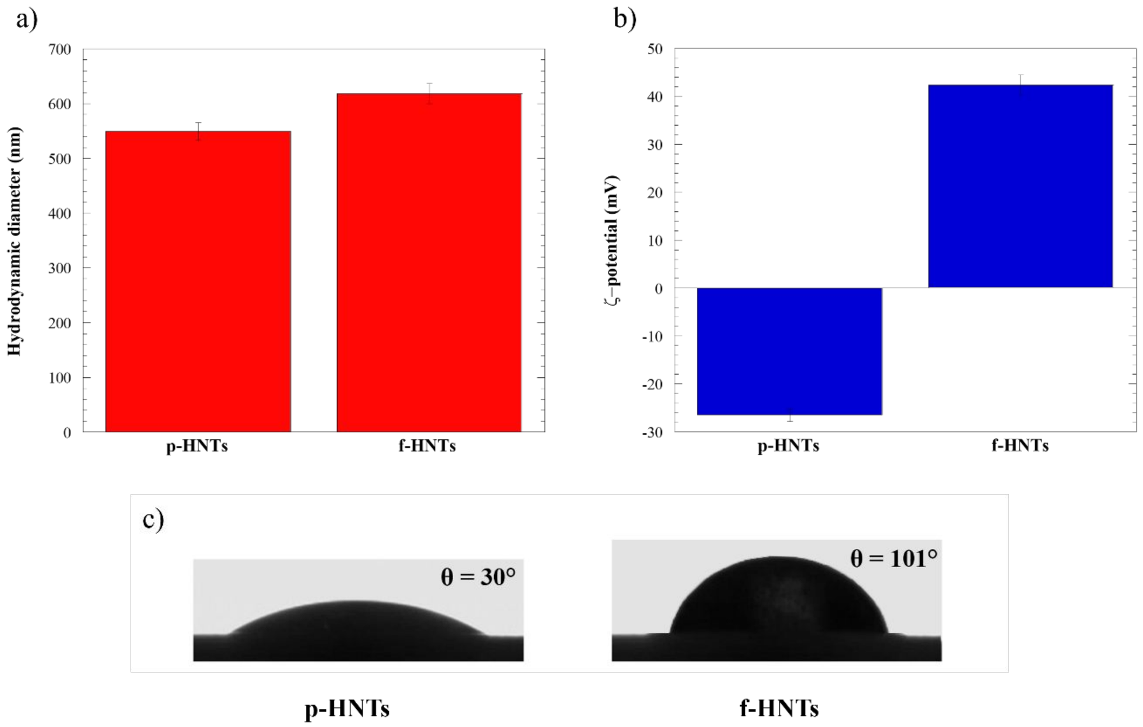

Figure S1. (a) Hydrodynamic diameters, (b) ζ-potential and (c) contact angle values for p-HNTs and f-HNTs.

Macroscopic and fluorescence microscopic images Pickering emulsions

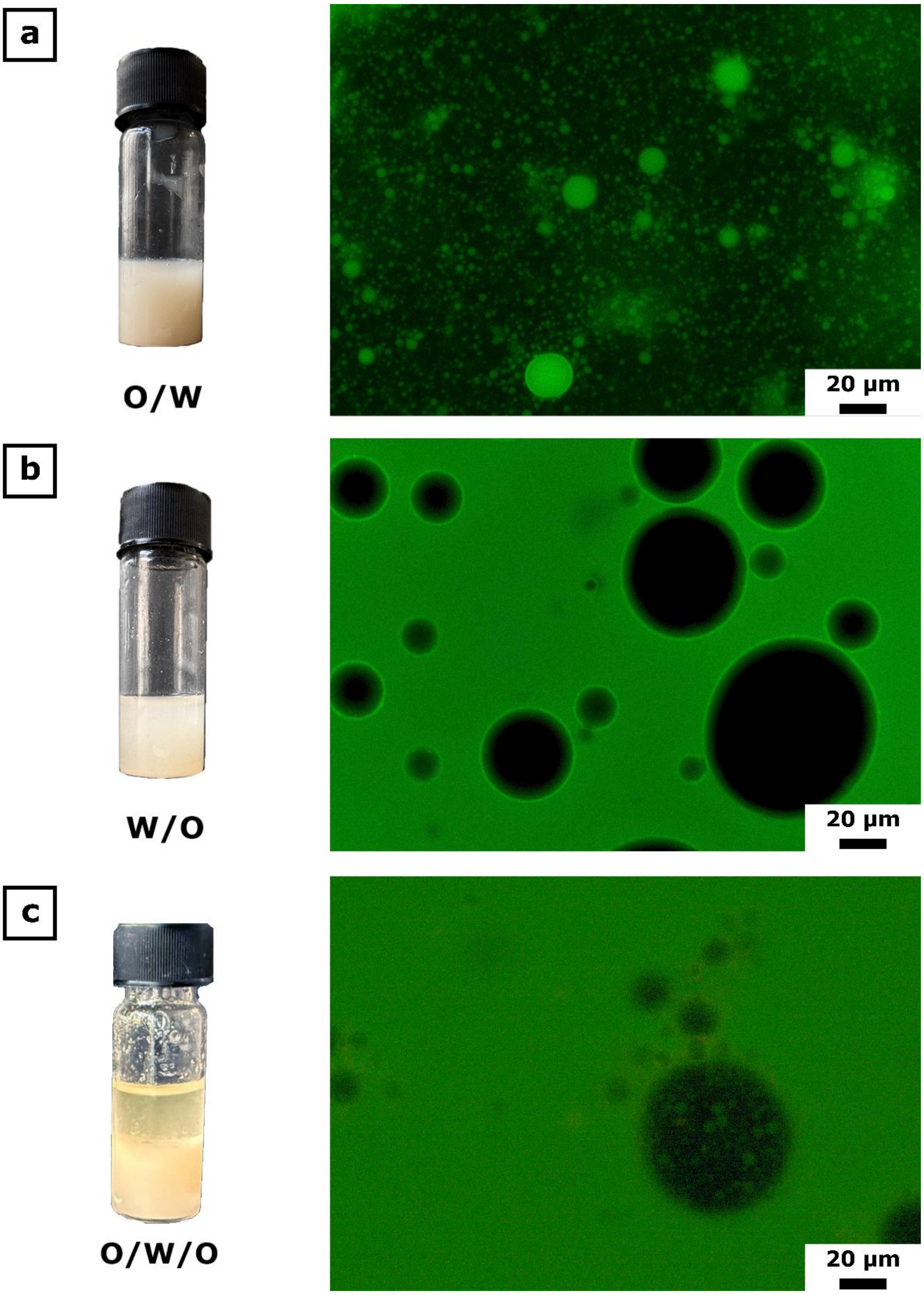

Figure S2. Macroscopic photos and fluorescence images of (a) o/w Pickering emulsions stabilized by p-HNTs; (b) w/o Pickering emulsions stabilized by f-HNTs; (c) o/w/o Pickering double emulsions.

Effect of the temperature on the o/w/o Pickering double emulsions

The thermal stability of the o/w/o Pickering double emulsions was evaluated by heating the sample from 20 to 60 ˚C. The temperature was increased in 10 ˚C increments (30, 40, 50, and 60 ˚C); at each step, the sample was maintained for 10 min before carrying out optical microscopy.

In particular, the system remained stable and the o/w/o Pickering double emulsions could be clearly observed at 20-40 ˚C (**Figure S3a-c**). Then, the droplets start to coalesce at 50 ˚C. At this condition, larger and less uniformly distributed Pickering double emulsions could be detected (Figure S3d). At 60 ˚C, complete phase separation occurred, and the characteristic o/w/o structures were no longer detectable (Figure S3e). Most importantly, the sample was allowed to cool down at room temperature and, upon rapid mixing, it was fully restored. The o/w/o Pickering double emulsions were still detectable and could be observed microscopically, proving that the temperature-induced destabilization process is reversible (Figure S3e).

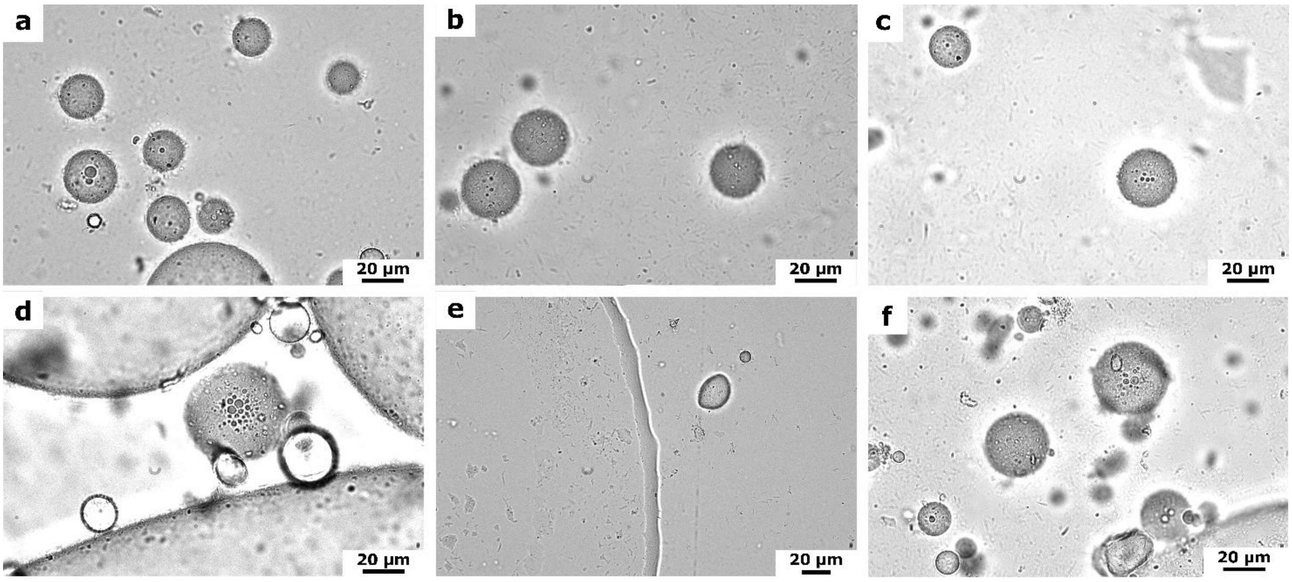

Figure S3. Optical microscopy images of o/w/o Pickering double emulsions at (a) 20 °C, (b) 30 °C (c) 40 °C, (d) 50 °C, (e) 60 °C and (f) after mixing upon cooling down.

Stability of the o/w/o Pickering double emulsions as a function of time

The stability of the o/w/o Pickering double emulsions over time was evaluated by monitoring the sample at fixed time intervals up to 24 h. At each time point, optical microscopy images were acquired (**Figure S4**) and subjected to statistical image analysis to assess droplet size distribution (**Figure S5**). This analysis was performed for both the internal o/w and the external w/o Pickering emulsions present within the double o/w/o system. As it can be observed, the dimensions of confined o/w Pickering emulsions were not affected over time and the radius of the droplets remained stable at 1.5-2 μm after 13 h. On the other side, both microscopy images and size distribution showed a clear tendency of the external w/o Pickering emulsions to coalesce into larger droplets over time. In particular, the average radius increased from approximately 20–40 μm to 40–100 μm after 13 h, indicating progressive droplet growth. After 24 h, complete phase separation was observed, with the loss of the characteristic o/w/o Pickering double emulsion structures. Importantly, as observed for the thermal stability study, the system could be restored by simple mixing. Upon re-homogenization, the emulsions became visible again, demonstrating that the time-dependent destabilization process is also reversible.

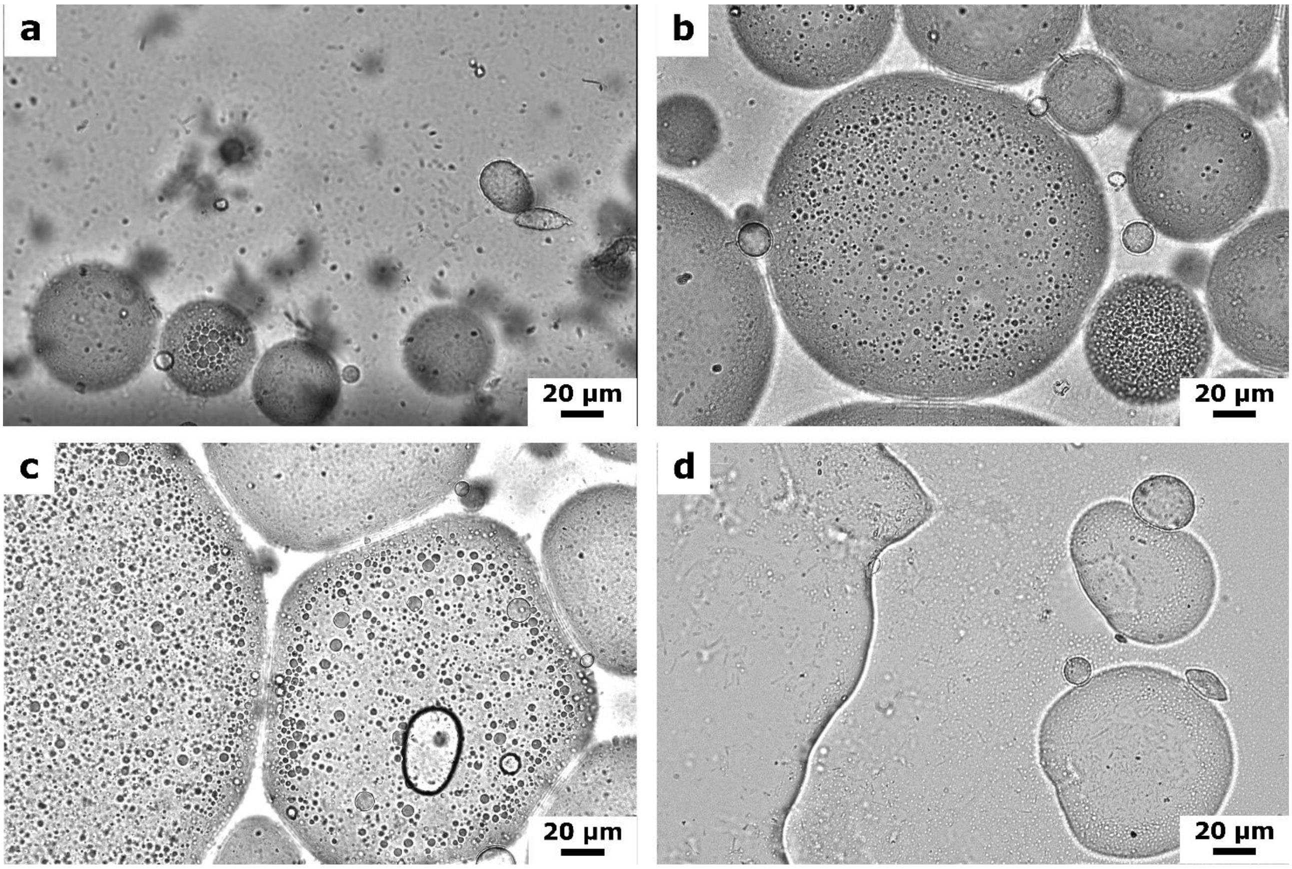

Figure S4. Optical microscopy images of o/w/o Pickering double emulsions (a) after preparation, (b) after 6 hours (c) after 13 hours and (d) after 24 hours.

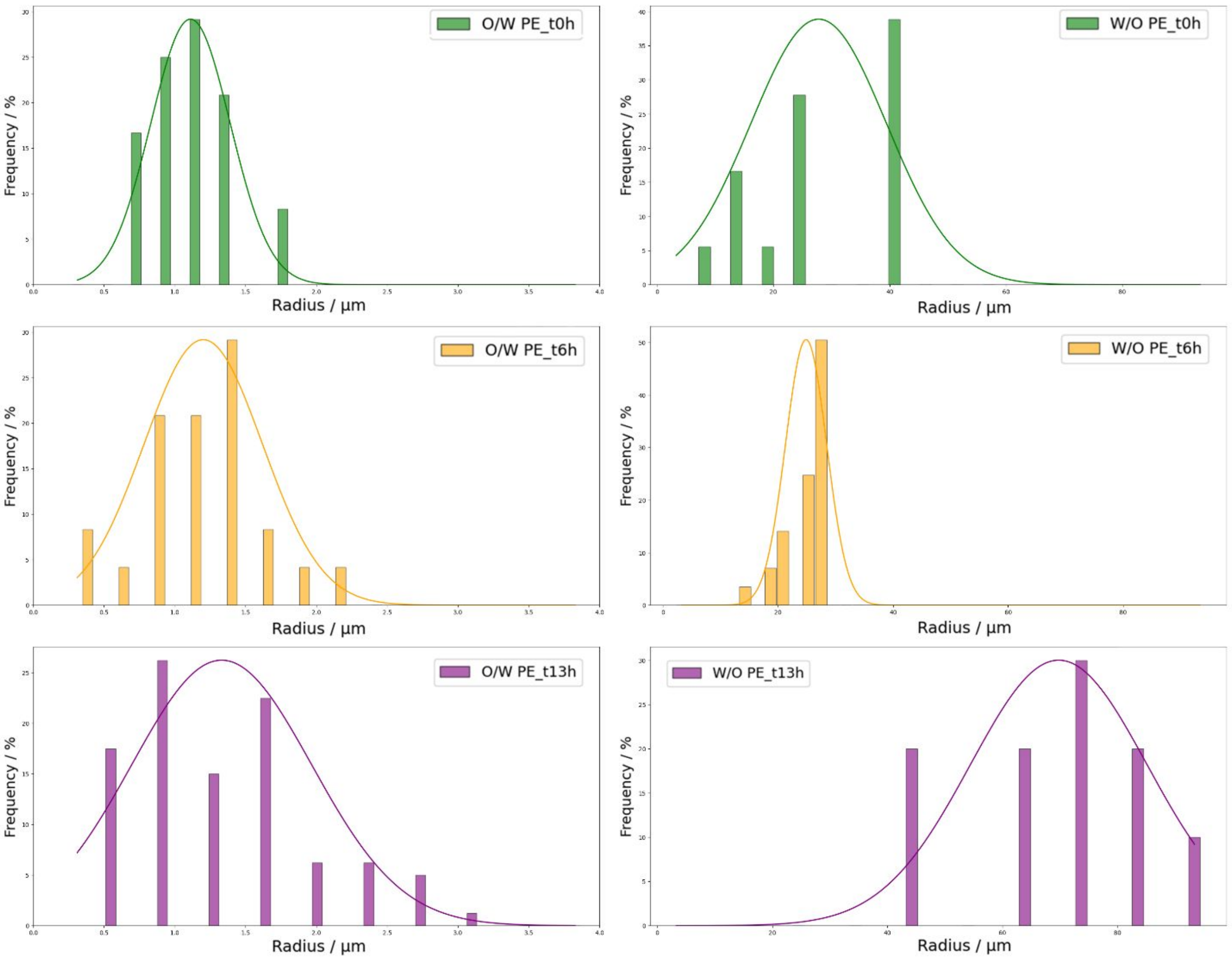

Figure S5. Size distribution of internal o/w and external w/o droplets in the o/w/o Pickering double emulsions.

Release of Nile Red from o/w/o Pickering double emulsions

The release profile of Nile red was investigated by UV-VIS spectrophotometry to assess whether the Pickering system can retain the dye more effectively due to its compartmentalization capability. Ethanol was used as release medium. Experiments were performed from o/w/o Pickering double emulsions and from a control solution of the dye in the bulk oil phase (i.e. decane). In the latter case, the dye was rapidly released. On the other side, it was observed that the presence of the hierarchical structure in the o/w/o Pickering double emulsions slowed the release kinetics in the ethanol medium, reaching 30% in 5 hours and 100% after 24 hours (**Figure S6**). These results reflect the dye localization within the oil droplets and validate the efficient compartmentalization capability of the material.

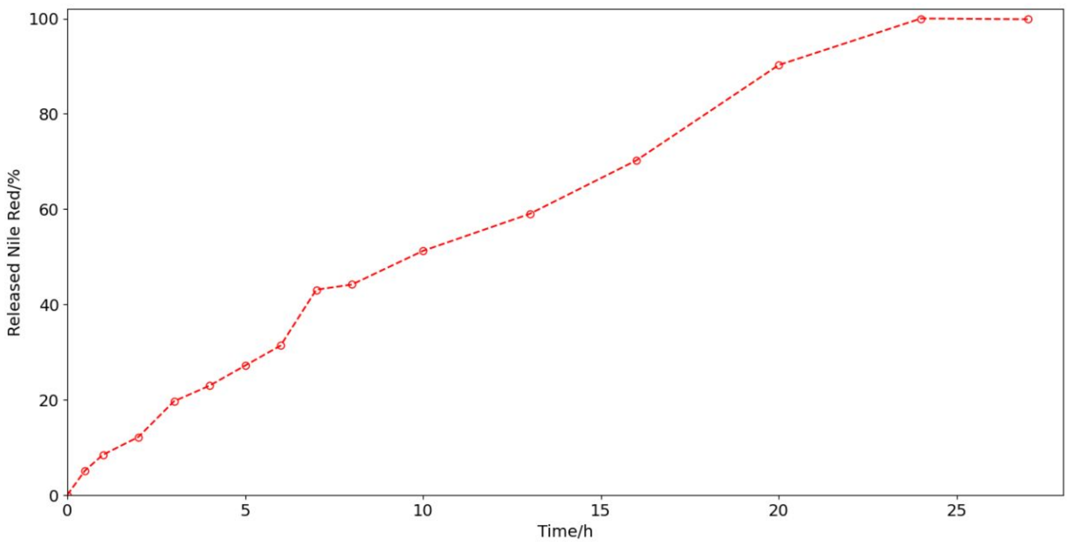

Figure S6. Release profile of Nile red from o/w/o Pickering double emulsions.

REFERENCES

(1) D'Agostino, G.; Petrasz, P.; Qing, W.; Zhou, H.; Stols-Witlox, M.; Bertrand, L.; Joseph, E.; Cavallaro, G.; Lazzara, G. From Inverse Pickering Emulsion to Polyhydroxybutyrate Gel Loaded with Chelators for Cleaning of Copper Surfaces: The Stabilization Effect of Hydrophobized Halloysite Clay Nanotubes. *J. Colloid Interface Sci.* **2026**, *703*, 139189. https://doi.org/10.1016/j.jcis.2025.139189.

(2) Cavallaro, G.; Lazzara, G.; Milioto, S.; Parisi, F. Hydrophobically Modified Halloysite Nanotubes as Reverse Micelles for Water-in-Oil Emulsion. *Langmuir* **2015**, *31* (27), 7472–7478. https://doi.org/10.1021/acs.langmuir.5b01181.

(3) Schneider, C. A.; Rasband, W. S.; Eliceiri, K. W. NIH Image to ImageJ: 25 Years of Image Analysis. *Nat. Methods* **2012**, *9* (7), 671–675. https://doi.org/10.1038/nmeth.2089.

(4) Tinevez, J.-Y.; Perry, N.; Schindelin, J.; Hoopes, G. M.; Reynolds, G. D.; Laplantine, E.; Bednarek, S. Y.; Shorte, S. L.; Eliceiri, K. W. TrackMate: An Open and Extensible Platform for Single-Particle Tracking. *Methods* **2017**, *115*, 80–90. https://doi.org/10.1016/j.ymeth.2016.09.016.
